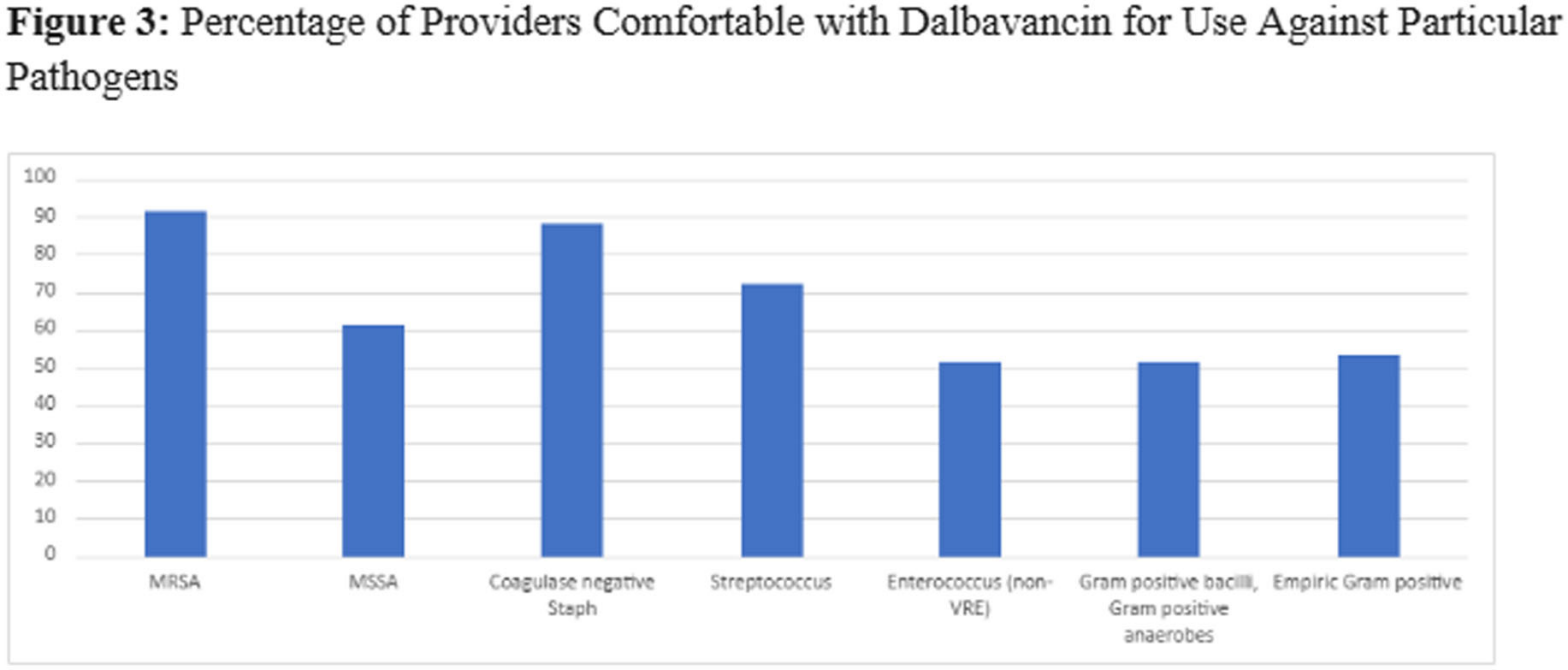# 110 National Simulation-Based Training Improves Central Venous Catheter Insertion and Maintenance Knowledge Across Israeli Hospitals

**DOI:** 10.1017/ash.2026.10527

**Published:** 2026-06-23

**Authors:** Michaela Barry, Judith Strymish, Andrew Chou, Duc Nguyen, Lauren Epstein

**Affiliations:** 1 Boston Medical Center; 2 VA Med Ctr - West Roxbury; 3 MEDVAMC & Baylor College of Medicine; 4 Atlanta VAMC

## Abstract

**Background:** Dalbavancin is increasingly used for management of serious gram-positive infections, even beyond those for which it has formal FDA approval. Barriers to dalbavancin prescription commonly faced in the private sector, such as high drug acquisition costs, insurance prior authorizations, and challenges coordinating infusions, are more easily managed within the VA system. Since dalbavancin was initially approved by the FDA in 2014, VA providers have prescribed approximately 11,500 doses nationally. We surveyed VA providers to understand their perceptions and experiences in using dalbavancin. **Methods:** We conducted a cross-sectional, 39 question survey via RedCap, which was distributed to approximately 400 VA infectious disease providers across 141 medical centers. The survey remained open from September 10 – November 10, 2025. **Results:** We received 82 responses from 63 (77%) infectious disease providers (physicians and advanced practice providers) and 18 (22%) pharmacists representing 50 unique VA facilities, spanning all 18 VA geographic regions. At nearly all facilities (48/50, 96%), dalbavancin was available and approval by an infectious disease specialist was required for use. While the median estimated dalbavancin use among VA providers was reported to be 1-2 prescriptions/month, there was wide variability in reported prescribing rates of dalbavancin overall (Figure 1). The majority of VA providers had used dalbavancin for skin and soft tissue infections, bloodstream infections, and osteoarticular infections. However, a minority reported use for endovascular infections. (Figure 2). Most providers perceived dalbavancin was efficacious against gram-positive organisms, excluding vancomycin resistant enterococci. (Figure 3). Half of providers reported drug cost as a barrier to prescribing dalbavancin (41/82, 50%), though few providers experienced objections or resistance from hospital leadership for dalbavancin prescription (14/80, 17%). Most providers (64/81, 78%) reported prescribing dalbavancin as a 2 dose regimen (doses 1 week apart). A minority (29/77, 38%) of participants felt it is important to obtain safety labs for patients receiving dalbavancin. Providers reported that the most important factors in choosing dalbavancin included ease of administration for the patient and concerns about non-adherence to other standard care therapies. **Conclusion:** The widespread administration of dalbavancin across the VA underscores its attractiveness as a treatment option within this system. It is often used beyond FDA-approved indications with wide variability in perceptions of effectiveness, depending on the pathogen. The lack of inclusion in the national VA formulary and the absence of formal stewardship guidelines highlight the need for future efforts. Notably, our survey was conducted following a recent clinical trial showing that dalbavancin is not inferior to other therapies for treating gram-positive infections. Developing stewardship guidelines and investigating experiences with dalbavancin outside the VA system is planned.

**Figure:**
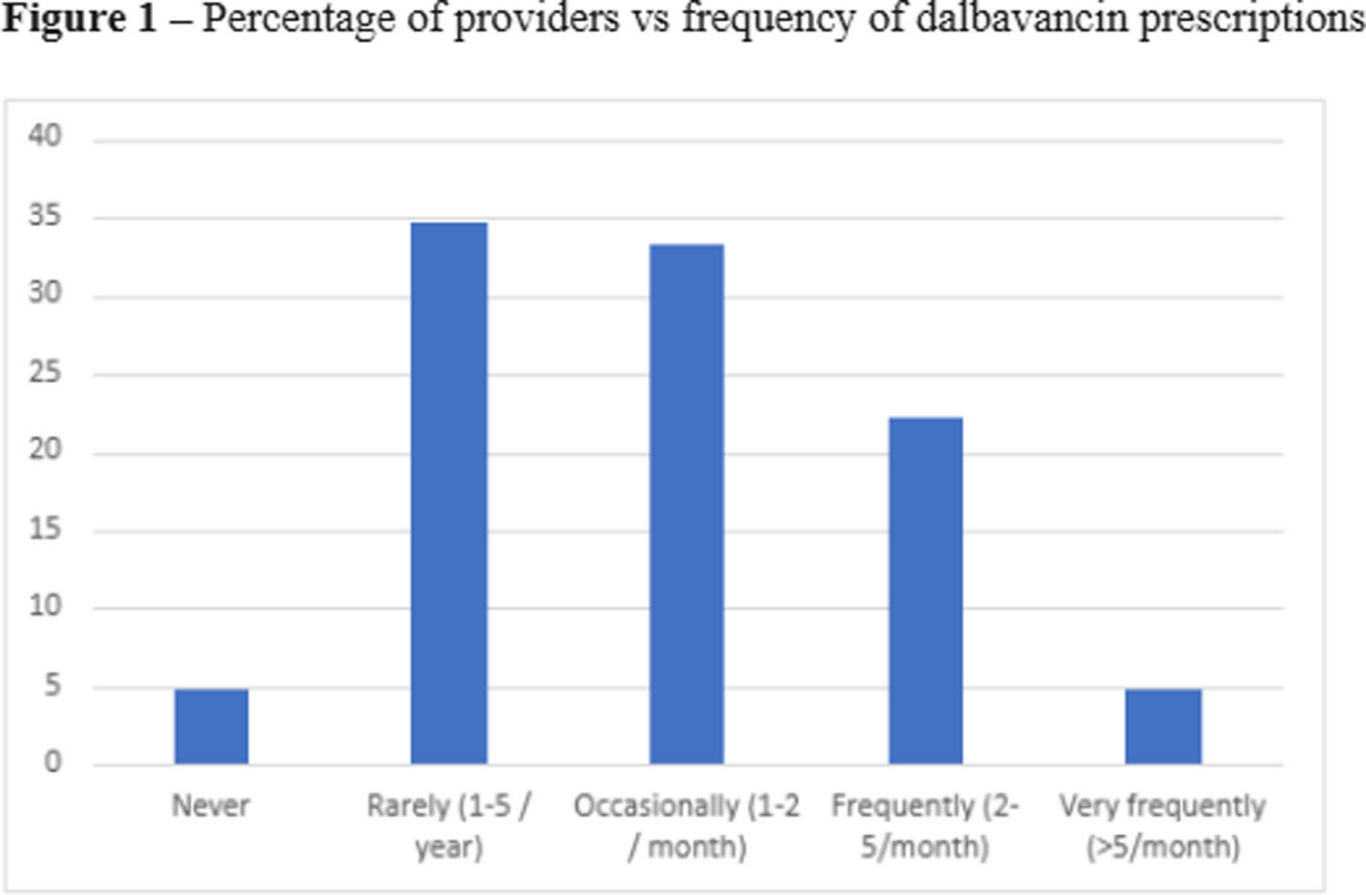

**Figure:**
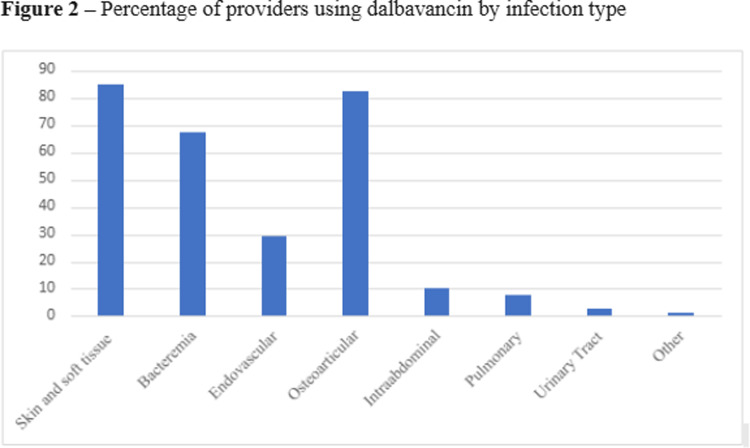

**Figure:**